# Logistic regression analysis of clinical and computed tomography features of pulmonary abscesses and risk factors for pulmonary abscess-related empyema

**DOI:** 10.6061/clinics/2019/e700

**Published:** 2019-04-02

**Authors:** Xing-Dong Cai, Ying Yang, Jinzhong Li, Xiaoying Liao, Shijie Qiu, Jingjing Xu, Miao Zhang, Yuanshun Huang, Zhi-Hong Huang, Hong-Ming Ma

**Affiliations:** I Department of Respiratory, The First Affiliated Hospital of Jinan University, Guangzhou 510630, China; II Health Management Center, The First Affiliated Hospital of Jinan University, Guangzhou 510630, China

**Keywords:** Pulmonary Abscess, Pulmonary Abscess-Related Empyema, Risk Factors

## Abstract

**OBJECTIVES:**

This study was conducted to investigate the risk factors for pulmonary abscess-related empyema by investigating the clinical characteristics and chest computed tomography imaging features of patients with pulmonary abscesses.

**METHODS:**

We retrospectively analyzed the chest computed tomography findings and clinical features of 101 cases of pulmonary abscess, including 25 cases with empyema (the experimental group) and 76 cases with no empyema (the control group). The potential risk factors for pulmonary abscess-related empyema were compared between the groups by using univariate and multivariate logistic regression analyses.

**RESULTS:**

The incidence of pulmonary abscess-related empyema was 24.8% (25/101). Univariate analysis showed that male gender, diabetes, pleuritic symptoms, white blood cells >10×10^9^/L, albumin level <25 g/L, and positive sputum cultures were potential clinical-related risk factors and that an abscess >5 cm in diameter and transpulmonary fissure abscesses were potential computed tomography imaging-related risk factors for pulmonary abscess-related empyema. Multivariate logistic regression analysis showed that transpulmonary fissure abscesses (odds ratio=9.102, *p*=0.003), diabetes (odds ratio=9.066, *p*=0.003), an abscess >5 cm in diameter (odds ratio=8.998, *p*=0.002), and pleuritic symptoms (odds ratio=5.395, *p*=0.015) were independent risk factors for pulmonary abscess-related empyema.

**CONCLUSIONS:**

Transpulmonary fissure abscesses, diabetes, giant pulmonary abscesses, and pleuritic symptoms increased the risk of empyema among patients with pulmonary abscesses.

## INTRODUCTION

Pulmonary abscesses are caused by pathogen infection and manifest as local necrotic and purulent lung tissue and cavity formation with a fluid level following pus drainage via a bronchial fistula 1,2. A pulmonary abscess is a serious respiratory tract infection resulting in mortality without antibiotic treatment in approximately one-third of affected cases. The mortality rate of pulmonary abscesses has been reduced to 8.7% as antibiotics have become widely used in clinical practice 3.

Pulmonary abscesses may be acute (<6 weeks) or chronic (≥6 weeks) and may be primary or secondary. Primary pulmonary abscesses, also known as aspiration pulmonary abscesses, are caused mainly by aspiration of nasopharyngeal secretions. Secondary pulmonary abscesses are secondary to bronchopulmonary disorders, including bronchial obstructive diseases (such as tumors, foreign bodies, and swollen lymph nodes), bronchopulmonary complications (such as bronchiectasis, pulmonary cystic fibrosis, and pneumonia), and infection of tissues located far from the lung or lung-surrounding tissues and spread to the lung via the bloodstream (such as infective endocarditis, cutaneous purulent disease, and subphrenic abscesses) 4. The risk factors for pulmonary abscesses include advanced age, dental or periodontal infections, drinking, drug abuse, diabetes, mechanical ventilation, epilepsy, neuromuscular disorders, malnutrition, therapy with glucocorticoids, cytotoxic drugs and immunosuppressants, mental retardation, gastroesophageal reflux, airway obstruction, inability to expectorate, and sepsis 5-7. Many pathogens can cause pulmonary abscesses, but primary pulmonary abscesses are mainly caused by gram-negative bacteria such as *Bacteroides fragilis*, *Clostridium perfringens*, and *Fusobacterium necrophorum* and Gram-positive anaerobic *Peptostreptococcus*. Secondary pulmonary abscesses are mainly caused by *Staphylococcus aureus*, *Streptococcus* spp., *Klebsiella pneumoniae*, *Pseudomonas aeruginosa*, *Haemophilus parainfluenzae*, *Acinetobacter* spp., and *Escherichia coli* 8-12.

Empyema is a serious and costly complication of pneumonia, with a mortality rate of up to 20% 13. The incidence of empyema is also high in patients with pulmonary abscesses. A study showed that among 387 patients with pulmonary abscesses and/or empyema, 15 (3.8%) had both conditions, and the most prevalent pathogen was *Streptococcus viridans* 14. Huang et al. 15 showed that among 259 patients with pulmonary abscesses, 22 (8.5%) also had empyema. The presence of empyema significantly increased the 30-day mortality and intensive care unit (ICU) admission rates of patients with pulmonary abscesses. For patients with pulmonary abscesses, the presence of empyema affects patient prognosis and requires different treatment strategies. This study was designed to investigate the risk factors for empyema in patients with pulmonary abscesses. We analyzed the differences in the clinical and chest computed tomography (CT) features of pulmonary abscess patients with or without empyema to identify major risk factors for pulmonary abscess-related empyema (PARE).

## MATERIALS AND METHODS

### Patients and risk factors

We collected and retrospectively analyzed 101 cases of pulmonary abscess treated at the First Affiliated Hospital of Jinan University between September 2011 and May 2018. Of these cases, 25 patients also had empyema confirmed by transthoracic aspiration and pleural effusion analysis. Written consent from patients and approval of the Institutional Research Ethics Committee of the First Affiliated Hospital of Jinan University were obtained. The patients were 17-87 years of age (mean: 54.9 years). Potential clinical-related risk factors included age (≤65 *vs* >65), gender (male *vs* female), smoking history, comorbidities, diabetes, malignant tumor, renal insufficiency (creatinine >106 µmol/L), inflammatory bronchial diseases (such as bronchiectasis, chronic bronchitis, and asthma), time from onset to admission, fever (>37.2°C), symptoms of pleuritis (pleuritic chest pain or anhelation), hemoptysis or bloody sputum, white blood cells (WBC)>10×10^9^/L (4-10×10^9^/L), anemia (hemoglobin [Hb]<11 g/dl), glycated hemoglobin ≤6.1%, serum albumin (<2.5 g/dl *vs* ≥2.5 g/dl), positive sputum culture (same pathogen identified by at least three consecutive sputum cultures with qualified sputum samples or same pathogen identified by both sputum culture and blood culture), clinical response (cure or improvement: disappearance of clinical symptoms and significant absorption or disappearance of the pulmonary abscess in chest CT images; no improvement: no absorption of the pulmonary abscess or empyema in chest CT images and incomplete absorption of the pulmonary abscess or empyema after surgical intervention, with improvement only of clinical symptoms), and the etiology of the lung abscess (primary *vs* secondary) 4 (Table 1). Risk factors on chest CT imaging were as follows: 1) the location of the pulmonary abscess; 2) the maximum diameter of the pulmonary abscess (>5 cm); 3) unilateral or bilateral multiple pulmonary abscesses: unilateral multiple pulmonary abscesses were defined as pulmonary abscesses in at least two lobes on the same side, and bilateral multiple pulmonary abscesses were defined as pulmonary abscesses in at least one lobe in each lung; 4) multilocular pulmonary abscess; 5) cavity formation with or without a fluid level; 6) PARE, which was defined as pleural empyema as a result of the spread or rupture of a pulmonary abscess (pleural effusion analysis showed WBC≥10×10^4^/L); empyema may have occurred after or concurrently with the pulmonary abscess and manifested as sudden chest pain and chest distress and/or dyspnea; 7) transpulmonary fissure abscess, which was defined as a pulmonary abscess that had spread to another lobe through a pulmonary fissure; 8) a thick-wall pulmonary abscess, which was defined as a pulmonary abscess whose wall was >5 mm in thickness; and 9) the edge of the pulmonary abscess (clear *vs* unclear) (Table 2).

Logistic regression analysis was performed to identify independent risk factors for PARE in which empyema was a dependent variable (Y:Y1=Yes, Y0=No) and other factors were independent variables (X1, X2, X3...). Significant variables (by χ^2^ test, *p*<0.01) were incorporated into the regression equation to identify independent risk factors for PARE.

### Statistical analysis

SPSS v16.0 (SPSS Inc., Chicago, IL, USA) was used for statistical analysis. Univariate analyses of all potential risk factors for PARE (categorical variables) were selected by using Pearson χ^2^ tests or Fisher's exact probability tests, and then the significant risk factors were incorporated into a multivariate logistic regression analysis to calculate odds ratios (ORs) and 95% confidence intervals (CIs). *p*<0.05 was considered statistically significant.

## RESULTS

### Clinical characteristics of patients with pulmonary abscesses

Among the 101 patients with pulmonary abscesses, 80 patients (79.2%, 80/101) were aged ≤65 years, 68 (67.3%, 68/101) were men, and 69 (68.3%, 69/101) were nonsmokers. For comorbidities, 33 patients (32.7%, 33/101) had diabetes, 29 of whom (87.9%, 29/33) had glycated hemoglobin >6.1%; 13 patients (12.9%) had malignant tumors; 16 patients (15.8%) had inflammatory bronchial diseases. Forty patients (39.6%, 40/101) were admitted to the hospital within one week of onset. Common symptoms were fever (46.5%), pleuritic symptoms (pleuritic chest pain or anhelation) (46.5%), and hemoptysis or bloody sputum (11.9%). Laboratory tests showed high WBC counts (57, 56.4%), anemia (35, 34.7%), hypoproteinemia (albumin <2.5 g/dl) (18, 17.8%), and positive sputum cultures (19, 18.8%; *Klebsiella pneumoniae*: n=7, *Acinetobacter baumannii*: n=5, *Staphylococcus aureus*: n=4, *Pseudomonas aeruginosa*: n=3, *Hemolytic streptococcus*: n=2, *Proteus mirabilis*: n=1, and *Escherichia coli*: n=1; mixed infection). Among the 101 patients with pulmonary abscesses, 5 patients (4.95%, 5/101) had positive blood cultures from venous blood samples drawn from the arms (*Staphylococcus aureus*: n=3 and *Klebsiella pneumoniae*: n=2); 5 (4.95%, 5/101) had positive pleural effusion cultures (*Streptococcus constellatus*: n=2, *Streptococcus viridans*: n=1, and *Acinetobacter lwoffii*: n=2) (Table 1).

### Chest CT imaging features of patients with pulmonary abscesses

**Figure 1:**
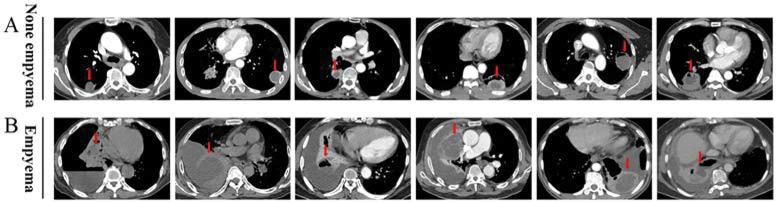
The chest CT image characteristics of pulmonary abscesses in patients without or with empyema A, Representative images from 76 cases of pulmonary abscess without empyema. B, Representative images from 25 cases of pulmonary abscess-related empyema. The arrow indicates a pulmonary abscess.

Among the 101 patients with pulmonary abscesses, 25 (24.8%, 25/101) had empyema. All cases were confirmed with chest CT, transthoracic aspiration, and pleural effusion analysis as needed (Figure 1). The pulmonary abscess was located in the lower lobes in 38 patients (37.6%), especially in the lower right lobe. Nineteen patients (18.81%) had unilateral multiple pulmonary abscesses; 14 (13.9%) had a pulmonary abscess in the upper right lobe; 60 (59.4%) had cavity formation; 46 (45.5%) had an abscess wall >5 mm; 42 (41.6%) had a pulmonary abscess >5 cm; 34 (33.7%) had a multilocular pulmonary abscess; 35 (34.7%) had multiple pulmonary abscesses; 30 (29.7%) had a pulmonary abscess with clear edges; and 18 (17.8%) had a transpulmonary fissure abscess (Table 2).

### Outcome of patients with pulmonary abscesses

Among the 101 patients with pulmonary abscesses, 93 (92.8%) responded to treatment and recovered. Pulmonary abscess patients with empyema were hospitalized for an average of 26.9 days, whereas those without empyema were hospitalized for an average of 24.1 days. Three patients (2.97%, 3/101) died. Five patients (4.9%, 5/101) did not respond to treatment, and 2 of these patients (40%, 2/5) had empyema after rupture of the pulmonary abscess.

### Risk factors for PARE

Univariate analysis showed that the clinical risk factors for PARE were male gender (*p*=0.04), diabetes (*p*=0.004), symptoms of pleuritis (pleuritic chest pain or anhelation) (*p*=0.003), WBC>10×10^9^/L (*p*=0.001), Albumin level <2.5 g/dl (*p*=0.012), and positive sputum culture (*p*=0.026) (Table 3). Chest CT risk factors for PARE were abscess diameter ≥5 cm (*p*<0.0001) and a transpulmonary fissure abscess (*p*<0.0001) (Table 4). Logistic regression analysis showed that a transpulmonary fissure abscess (OR=9.102, *p*=0.003), diabetes (OR=9.066, *p*=0.003), an abscess >5 cm in diameter (OR=8.998, *p*=0.002), and pleuritic symptoms (OR=5.395, *p*=0.015) were independent risk factors for PARE (Table 5).

## DISCUSSION

Empyema as a result of the rupture or spread of a pulmonary abscess not only prolongs the hospital stay but also increases mortality. This study showed that the incidence of empyema was 24.8% (25/101) in patients with a pulmonary abscess. Among the 101 patients with a pulmonary abscess, 92.8% responded to anti-infective therapy alone, consistent with the findings of Erasmus et al. 16, who found that most patients required no surgical intervention for a pulmonary abscess. Among the 5 patients whose condition did not improve as a result of poor response to treatment, 2 had PARE and required surgical intervention, indicating that empyema was the leading cause of surgical intervention in patients with a pulmonary abscess.

Pulmonary abscesses may occur in any age group 17. This study showed that 79.2% of patients with a pulmonary abscess were ≤65 years of age, especially those who were male (67.3%), consistent with literature reports 6,18. These findings indicate that men and younger populations are high-risk groups for pulmonary abscesses. Pulmonary abscesses present no specific manifestations. Most patients have fever, chest pain, chest distress, and dyspnea; some have hemoptysis or bloody sputum, according to literature reports 18. Pulmonary abscesses are caused mainly by pathogen infection. Takayanagi et al. 9 retrospectively analyzed 205 cases of community-acquired pulmonary abscesses and found that *Streptococcus* spp. (59.8%), anaerobic bacteria (26.2%), gemella species (9.8%), and *Klebsiella pneumoniae* (8.2%) were the major pathogens. In the present study, sputum and blood cultures identified *Klebsiella pneumoniae*, *Acinetobacter baumannii, Staphylococcus aureus*, *Hemolytic streptococcus*, *Proteus mirabilis*, *Escherichia coli*, and *Pseudomonas aeruginosa* as the major pathogens, especially in patients with empyema, which was the result of spread via the lymphatic system or direct spread to the parietal pleura 19. 

Approximately 75% of pulmonary abscesses occur in the posterior segment of the right upper lobe and in the dorsal segment of the lower lobe 3, whereas aspiration pulmonary abscesses usually occur in the posterior or dorsal segment, and blood-borne pulmonary abscesses may occur in any segment. This study showed that 50 cases (49.5%) of pulmonary abscess occurred in the right lung, and 38 (37.6%) occurred in both lower lobes, suggesting that pulmonary abscesses may be associated with aspiration of airway secretions, consistent with literature reports 3. Moreover, 35 patients (34.7%) had unilateral or bilateral multiple pulmonary abscesses, and the incidence was higher than that in literature reports (3.2%) 3, suggesting that the incidence of multiple pulmonary abscesses may be increasing (Table 3). In addition, this study showed that 42 patients (41.6%) had a pulmonary abscess with a maximum diameter of ≥5 cm. For pulmonary abscesses with cavity formation, the liquefied and necrotic tissue is eliminated via the bronchi, resulting in the formation of a cavity with or without a fluid-gas level. This study showed that 60 patients (59.4%) had a cavity, and the remaining patients exhibited liquefaction and necrosis without a cavity. Among the 25 patients with PARE, 17 had cavities, suggesting that gas-filled abscesses were more likely to rupture or spread to the pleural cavity to form empyema. It should be noted that in this study, 25 of the 101 patients (24.8%) had PARE, which significantly prolonged their hospital stay and affected the treatment outcome. Most of these cases required surgical intervention, suggesting that it is important to prevent empyema in patients with pulmonary abscesses to reduce hospital stays and improve patient outcomes.

The risk factors for pulmonary abscesses were advanced age, dental or periodontal disease, drinking, drug abuse, diabetes, smoking, coma, mechanical ventilation, epilepsy, neuromuscular disorders with bulbar palsy, malnutrition, therapy with glucocorticoids, cytotoxic drugs and immunosuppressants, mental retardation, gastroesophageal reflux, airway obstruction, inability to expel sputum, sepsis, and positive sputum culture such as *Pseudomonas aeruginosa* 5,7,20. The risk factors for an adverse prognosis of pulmonary abscesses were giant pulmonary abscesses (cavity >6 cm), advanced age, neoplasm, confusion, and bacterial infection with *Klebsiella pneumoniae*, *Pseudomonas aeruginosa*, or *Staphylococcus* spp. 21. This study was the first to investigate the risk factors for PARE. Univariate analysis showed that the clinical-related risk factors for PARE were male gender (*p*=0.04), diabetes (*p*=0.004), pleuritic symptoms (*p*=0.003), white blood cells (WBC)>10×10^9^/L (*p*=0.001), albumin level <2.5 g/dl (*p*=0.012), and positive sputum or blood culture (*p*=0.026) (Table 3). Diabetes is a risk factor for pulmonary abscesses, adverse prognosis and PARE. This study showed that among the 33 pulmonary abscess patients with diabetes, 87.9% had uncontrolled glycosylated hemoglobin, suggesting that diabetes, especially uncontrolled blood sugar, is a risk factor for PARE. Furthermore, for patients with pulmonary abscesses, pathogens such as *Klebsiella pneumoniae* and *Staphylococcus aureus* are more aggressive and destructive to lung tissue 22,23 and are more likely to invade the visceral pleura, even causing the rupture of the pulmonary abscess and ensuing empyema. In addition, this study showed that the risk of empyema was higher in pulmonary abscess patients with chest distress and/or dyspnea, indicating that invasion of the pleura by the pulmonary abscess increased the susceptibility to empyema. Pulmonary abscess patients with an albumin level <2.5 g/dl also had an increased risk of empyema, whereas age, smoking, tumor, anemia, hemoptysis or bloody sputum and the etiology of the lung abscess (primary *vs* secondary) were not risk factors for PARE.

Giant pulmonary abscesses are associated with an adverse prognosis. This study showed that for patients with pulmonary abscesses ≥5 cm, the risk of empyema was significantly higher than for those with smaller pulmonary abscesses (OR=8.998, 95% CI: 2.18-37.09, *p*=0.002). In addition, transpulmonary fissure abscesses had the highest ORs (OR=9.102, 95% CI: 2.09-36.65, *p*=0.003) in the multivariate logistic analysis and were a major risk factor for PARE. The horizontal and oblique fissures that separate the corresponding lobes are dense; hence, the presence of a pulmonary abscess that breaks through the horizontal or oblique fissures indicates aggressive infection that is prone to spreading, result in destruction of the surrounding tissue and empyema once the infection has spread to the pleural cavity. Furthermore, multivariate logistic regression analysis showed that diabetes mellitus and pleuritic symptoms were also independent risk factors for PARE (Table 5), suggesting that clinicians should remain vigilant about PARE in patients with these risk factors.

This is a retrospective analysis with some limitations. First, this was a single-center study with a small sample size, which may result in selection bias. Thus, large sample studies are needed to validate the results. Second, PARE was determined based on chest CT images, transthoracic aspiration, and pleural effusion analysis. We did not perform pleural biopsy near the pulmonary abscess; thus, we were unable to determine whether empyema was caused by rupture or spread of the pulmonary abscess.

## AUTHOR CONTRIBUTIONS

Cai XD and Yang Y were responsible for the study conception and design. Li J, Liao X, Qiu S, Xu J, Zhang M and Huang Y were responsible for the data acquisition. Cai XD, Huang ZH, Ma HM were responsible for the data analysis and interpretation. Cai XD and Yang Y were responsible for the manuscript drafting. Cai XD and Ma HM were responsible for the manuscript critical revision.

## Figures and Tables

**Table 1:** The clinical characteristics of 101 patients with pulmonary abscesses.

Clinical characteristics	n (%)
Age (years)	
≤65	80 (79.2)
>65	21 (20.8)
Gender	
Male	68 (67.3)
Female	33 (32.7)
Smoking	
Yes	32 (31.7)
No	69 (68.3)
Diabetes mellitus	
Yes	33 (32.7)
No	68 (67.3)
Malignant tumor	
Yes	13 (12.9)
No	88 (87.1)
Inflammatory bronchial disease	
Yes	16 (15.8)
No	85 (84.2)
Renal insufficiency	
Yes	11 (10.9)
No	90 (89.1)
Time from onset to admission	
≤1 week	40 (39.6)
1∼2 weeks	24 (23.8)
>2 weeks	37 (36.6)
Fever	
Yes	47 (46.5)
No	54 (53.5)
Occurrence of pleuritic symptoms	
Yes	47 (46.5)
No	54 (53.5)
Hemoptysis or bloody sputum	
Yes	12 (11.9)
No	89 (88.1)
White blood cell count (×10^9^/L)	
≤10	44 (43.6)
>10	57 (56.4)
Hemoglobin (g/dl)	
<11	35 (34.7)
≥11	66 (65.3)
Glycated hemoglobin (%)	
≤6.1	4/33 (12.1)
>6.1	29/33 (87.9)
Albumin level (g/dl)	
<2.5	18 (17.8)
2.5∼3.5	49 (48.5)
>3.5	34 (33.7)
Pathogenic bacteria in sputum	
Positive	19 (18.8)
Negative	71 (70.3)
No sputum	11 (10.9)
Etiology of the pulmonary abscess	
Primary	74 (73.3)
Secondary	27 (26.7)
Clinical outcome	
Death	3 (2.97)
Not improved	5 (4.9)
Improved or cured	93 (92.08)

**Table 2:** Chest CT imaging features of 101 patients with pulmonary abscesses.

Chest CT imaging features	n (%)
Pulmonary abscess location	
Right upper lobe	14 (13.9)
Right middle lobe	2 (1.98)
Right lower lobe	23 (22.77)
Left upper lobe	12 (11.88)
Left lower lobe	15 (14.85)
Bilateral pulmonary multiple lesions	16 (15.84)
Unilateral pulmonary multiple lesions	19 (18.81)
Maximum diameter of the pulmonary abscess	
≥5 cm	42 (41.6)
<5 cm	59 (58.4)
Multiple pulmonary abscesses	
Yes	35 (34.7)
No	66 (65.3)
Multilocular pulmonary abscess	
Yes	34 (33.7)
No	67 (66.3)
Cavity formation of pulmonary abscess	
Yes	60 (59.4)
No	41 (40.6)
Pulmonary abscess-related empyema	
Yes	25 (24.8)
No	76 (75.2)
Transpulmonary fissure abscess	
Yes	18 (17.8)
No	83 (82.2)
Wall thickness of the pulmonary abscess	
>5 mm	46 (45.5)
≤5 mm	55 (54.5)
Clear edge of the pulmonary abscess	
Yes	30 (29.7)
No	71 (70.3)

**Table 3:** Univariate analysis of the correlation between PARE and clinical risk factors.

Variables	n	Empyema	χ^2^	*p*
Age (years)				
≤65	80	18	1.05	0.31
>65	21	7		
Gender				
Male	68	21	4.199	0.04
Female	33	4		
Smoking				
Yes	32	8	0.002	0.97
No	69	17		
Diabetes mellitus				
Yes	33	14	8.218	0.004
No	68	11		
Malignant tumor				
Yes	13	2	0.703	0.402
No	88	23		
Inflammatory bronchial diseases				
Yes	16	2	0.85	0.356
No	85	23		
Renal insufficiency				
Yes	11	2	0.286	0.593
No	90	23		
Time from onset to admission				
<1 week	40	11	4.76	0.092
1∼2 weeks	24	9		
>2 weeks	37	5		
Fever				
Yes	47	15	2.42	0.12
No	54	10		
Pleuritic symptoms				
Yes	47	18	8.66	0.003
No	54	7		
Hemoptysis or bloody sputum				
Yes	12	1	1.971	0.16
No	89	24		
White blood cell count (×10^9^/L)				
≤10	44	5	11.095	0.001
>10	57	20		
Hemoglobin (g/dl)				
<11	35	8	0.103	0.748
≥11	66	17		
Albumin level (g/dl)				
<2.5	18	9	6.989	0.012
≥2.5	47	16		
Pathogenic bacteria in sputum				
Positive	19	9	7.305	0.026
Negative	71	15		
No sputum	11	1		
Etiology of the lung abscess				
Primary	74	20	0.769	0.381
Secondary	27	5		

**Table 4:** Univariate analysis of the correlation between PARE and chest CT imaging risk factors.

Variables	n	Empyema	χ^2^	*p*
Pulmonary abscess location				
Right upper lobe	14	1	8.361	0.203
Right middle lobe	2	0		
Right lower lobe	23	8		
Left upper lobe	12	1		
Left lower lobe	15	5		
Bilateral pulmonary multiple lesions	16	3		
Single pulmonary multiple lesions	19	7		
Maximum diameter of the pulmonary abscess				
≥5 cm	42	18	12.653	<0.0001
<5 cm	59	7		
Multiple pulmonary abscesses				
Yes	35	10	0.419	0.517
No	66	15		
Multilocular pulmonary abscess				
Yes	34	12	3.058	0.08
No	67	13		
Cavity formation of the pulmonary abscess				
Yes	60	17	1.018	0.313
No	41	8		
Transpulmonary fissure abscess				
Yes	18	11	15.546	<0.0001
No	83	14		
Wall thickness of the pulmonary abscess				
>5 mm	46	8	2.458	0.117
≤5 mm	55	17		
Clear edge of the pulmonary abscess				
Yes	30	7	0.046	0.83
No	71	18		

**Table 5:** Logistic regression analysis of clinical and chest CT imaging risk factors for PARE.

Variables	B	SE	Wald	OR (95% CI)	*p*
Transpulmonary fissure abscess					
Yes	2.209	0.751	8.654	9.102 (2.09-36.65)	0.003
No				1.00	
Diabetes mellitus					
Yes	2.204	0.734	9.014	9.066 (2.15-38.23)	0.003
No				1.00	
Abscess >5 cm in diameter					
>5 cm	2.197	0.723	9.245	8.998 (2.18-37.09)	0.002
≤5 cm				1.00	
Pleuritic symptoms					
Yes	1.686	0.692	5.938	5.395 (1.39-20.93)	0.015
No				1.00	
White blood cell count (×10^9^/L)					
>10	0.691	0.717	0.929	1.996 (0.49-8.15)	0.335
≤10				1.00	
